# Hypercalcemia affected in metastatic breast cancer patients without bone metastasis: report of three cases

**DOI:** 10.1186/s40792-022-01565-9

**Published:** 2022-11-26

**Authors:** Sakina Hashizume, Masafumi Shimoda, Tetsuhiro Yoshinami, Kaori Abe, Nanae Masunaga, Yoshiaki Sota, Tomohiro Miyake, Tomonori Tanei, Kenzo Shimazu

**Affiliations:** Department of Breast and Endocrine Surgery, Osaka University Graduate School of Medicine, 2-2-E10 Yamadaoka, Suita, Osaka 565-0871 Japan

**Keywords:** Humoral hypercalcemia of malignancy, Breast cancer, PTHrP, Local osteolytic hypercalcemia

## Abstract

**Background:**

Since humoral hypercalcemia of malignancy (HHM) in breast cancer patients without bone metastasis is rare, the clinical features of this condition are not fully understood.

**Case presentation:**

During the recent 12 years, 3602 patients were diagnosed with breast cancer in our institution, and only three patients developed HHM without bone metastasis. They were all recurrent breast cancer patients with visceral metastases including the lung and the liver. It took no more than 2 months since symptomatic onset to hospitalization because of hypercalcemia. The maximum serum calcium concentrations were 15.0 mg/dL or higher. All patients had symptoms related to hypercalcemia. Treatment of hypercalcemia including hydration, calcitonin, bisphosphonate, and diuretics was initially effective in the three patients. However, two of three cases were eventually fatal because of unsuccessful treatment of breast cancer.

**Conclusions:**

The common features of HHM without bone metastasis in breast cancer patients include acute onset, severe symptomatic hypercalcemia, and presence of visceral metastasis. Treatment of hypercalcemia decreased serum calcium level in a short period, while successful treatment of breast cancer was essential for a long-term management of HHM. This report provides a consideration to help elucidate the pathophysiology and medical care of breast cancer patients with HHM without bone metastasis.

## Background

Up to 30% of cancer patients experience hypercalcemia, which is a common complication [1] usually caused by parathyroid hormone-related protein (PTHrP)-induced humoral hypercalcemia of malignancy (HHM), local osteolytic hypercalcemia (LOH) associated with bone metastasis, or both. The overproduction or intoxication of 1,25-dihydroxy vitamin D and accompanying primary hyperparathyroidism is less common, but needs to be considered.

The leading cause of hypercalcemia associated with malignancy is HHM, which is accounted for up to 80% of hypercalcemia of malignancy [2]. HHM is induced by overproduction of PTHrP, a hormone similar to parathyroid hormone (PTH, encoded by the *PTH* gene), in tumor cells. PTHrP is a small protein encoded by *PTHLH* gene and increases the serum calcium level by acting on the bone and the kidney; PTHrP activates osteoblastic cells in the bone through PTH receptor 1 to promote bone resorption and promotes distal tubular calcium resorption in the kidney. This oncologic emergency rapidly progresses and eventually becomes fatal despite the availability of HHM treatments [3]. On the other hand, LOH occurs when tumor cells in the bone produce paracrine factors such as tumor necrosis factor (TNF) α and interleukin-1, which in turn hyperactivate osteoclasts and enhance bone resorption in patients with extensive bone metastasis.

In patients with metastatic breast cancer, LOH is the most prevalent cause of hypercalcemia, because almost all metastatic breast cancer patients with hypercalcemia have bone metastasis. HHM is frequently observed in patients with squamous cell carcinoma, renal cell carcinoma, and ovarian carcinoma, while breast cancer rarely (< 1%) manifests HHM without bone metastasis [4]. Therefore, HHM’s clinicopathological features without bone metastasis in breast cancer patients are not fully understood. Here, we report three HHM cases with no bone metastasis among 3602 cases diagnosed with breast cancer treated in our institution. All 3602 cases were reviewed for bone metastases. Along with five cases reported previously, the common clinical pictures of HHM without bone metastasis in patients with breast cancer are discussed in the present report.

## Case presentation

### Case 1

**Fig. 1:**
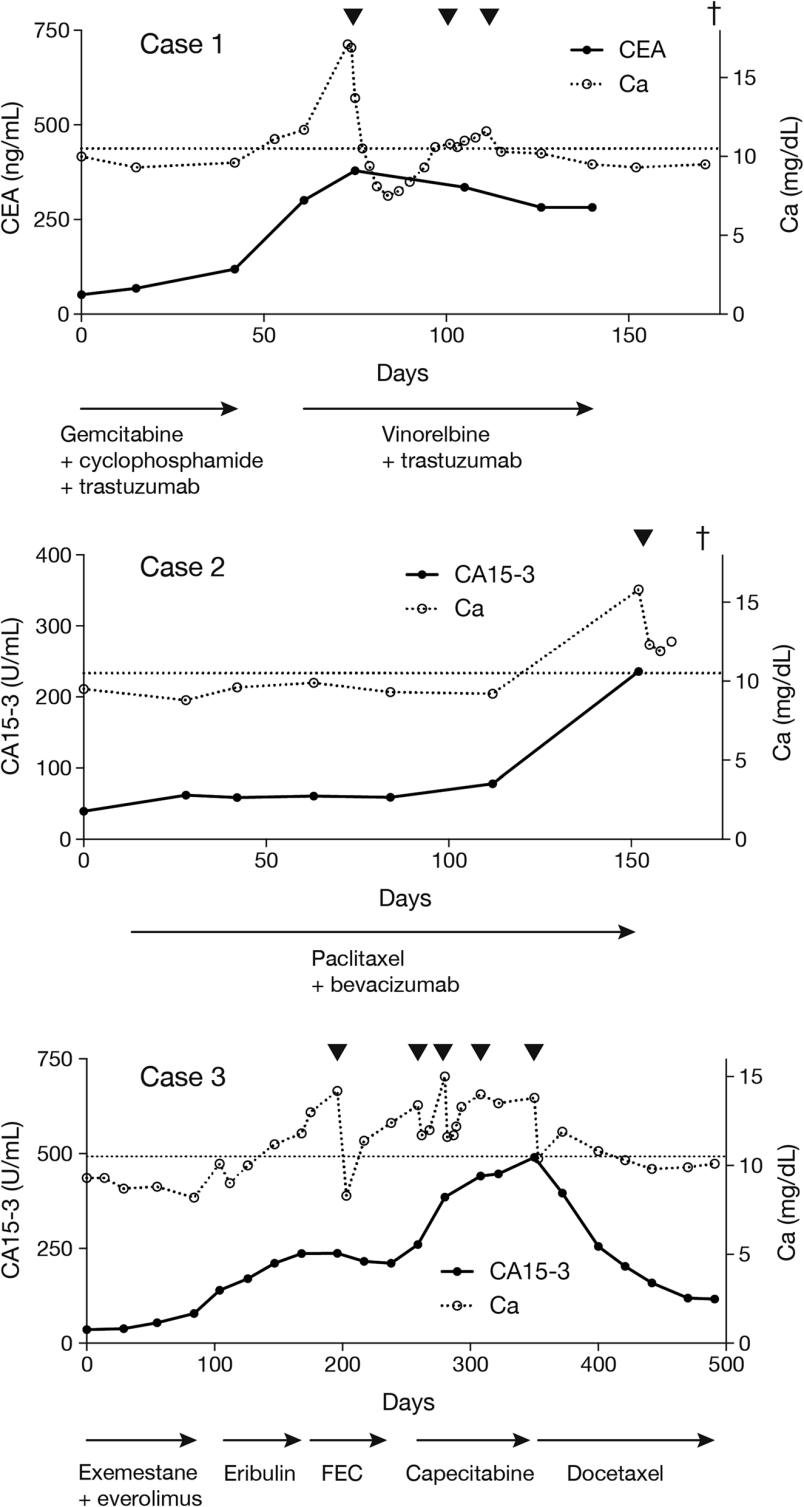
Time course of serum calcium and CA15-3 levels in Cases 1–3. Black triangles denote administration of zoledronate. A cross represents death. A horizontal broken line indicates the upper limit of serum calcium concentration. *FEC* fluorouracil + epirubicin + cyclophosphamide

A 43-year-old woman with right breast cancer T2N1M0 received nipple-sparing mastectomy and axillary lymph node dissection. The tumor was estrogen receptor (ER)-positive and human epidermal growth factor receptor-2 (HER2)-positive. Recurrence in the preserved nipple occurred 10 years after the initial surgery, and local resection was performed. Three years later, recurrence was observed in the lung and the pleura. Chemotherapy induced clinical complete remission, but new liver metastasis appeared 1 year later. At that time, serum calcium mildly increased to 11.5 mg/dL, but then rapidly increased to 17.1 mg/dL after the administration of vinorelbine and trastuzumab as the seventh-line treatment. She developed nausea and altered mental status. No bone metastasis was found in the latest computed tomography (CT) scan. Serum PTH and PTHrP were 10.4 pg/mL and 153 pmol/L, respectively, indicating the presence of HHM. Zoledronate infusion, hydration and calcitonin administration were initially effective. Zoledronate was administered twice to continually control hypercalcemia after terminating calcitonin administration. Although serum calcium was controlled thereafter, she died of respiratory failure 4 months after the onset of HHM. The time course of serum calcium and CEA levels is shown in Fig. 1.

### Case 2

A 39-year-old woman with right breast cancer T3N1M0 underwent mastectomy and axillary lymph node dissection, followed by chemotherapy, radiotherapy, and endocrine therapy. Her breast tumor was ER-positive and HER2-negative. Two years and 8 months after surgery, she suffered from recurrent breast cancer in the liver. After 4 years and 7 months since the recurrence, she started to receive paclitaxel and bevacizumab as the tenth-line treatment against metastatic breast cancer. Five months later, metastatic tumors in the liver rapidly progressed, and she developed mouth dryness and abdominal pain. As serum calcium rose to 15.5 mg/dL, she was hospitalized. Hydration, diuretics, and calcitonin administration followed by zoledronate administration improved serum calcium level to 10.9 mg/dL on the 7th day of hospitalization. She died of liver failure on the 18th day. Bone metastasis as well as parathyroid swelling was not observed in CT and positron emission tomography–CT scans, clinically suggesting the presence of HHM, though PTH and PTHrP were not measured. The time course of serum calcium and CA15-3 levels is shown in Fig. 1.

### Case 3

A 60-year-old woman with right breast cancer T2N1M0 received lumpectomy and axillary lymph node dissection after neoadjuvant chemotherapy. Her breast tumor was ER-positive and HER2-negative. She underwent postoperative radiotherapy and endocrine therapy, but 1 year later, multiple lung and liver metastases occurred. Endocrine treatment and chemotherapy against metastatic breast cancer was continued for 4 years and 8 months. Pulmonary lesions disappeared after a short time since the treatment and did not relapse. During the fifth-line treatment with eribulin, serum calcium and tumor markers including CEA and CA15-3 gradually increased. Eribulin was discontinued and zoledronate was administered with significant serum calcium decrease. Then fluorouracil + epirubicin + cyclophosphamide was administered but did not alleviate the hypercalcemia. After repeat zoledronate administration, capecitabine was started as anticancer therapy. Two months later, hypercalcemic symptoms such as dry mouth and altered state of consciousness appeared. CT and positron emission tomography–CT scans revealed further exacerbation of liver metastasis. Bone was not affected. Serum calcium increased to 14.2 mg/dL, and PTHrP also increased to 16.7 pmol/L, indicating the presence of HHM. She was hospitalized and started to receive hydration and calcitonin injections followed by zoledronate administration. Symptoms gradually disappeared, but the serum calcium level was still as high as 13.8 mg/dL. Capecitabine was discontinued, and docetaxel administration once every 3 weeks began, which was effective in suppressing the hepatic lesions. In accordance with the reduction of tumor load, serum calcium level was also decreased to the normal level without any treatments for hypercalcemia. The treatment with docetaxel is ongoing. The time course of serum calcium and CA15-3 is shown in Fig. 1.

## Discussion

LOH can be sometimes encountered in patients with bone metastasis of breast cancer. The serum calcium level is often mildly elevated (10.5–11.9 mg/dL), and the patients are generally asymptomatic. Treatment of mild hypercalcemia is not required in many cases. Such mild clinical presentation of LOH may be because denosumab or zoledronic acid, both of which are a standard-of-care for hypercalcemia, has already been used to prevent pathologic fractures of the metastasized bones.

In contrast to LOH, HHM prevalence without bone metastasis is less than 1% in patients with metastatic breast cancer, and thus, the clinical picture and the response to treatment in breast cancer patients are not fully understood. Only three cases of HHM without bone metastasis were found among 3602 cases with breast cancer diagnosed in our institution between January 2010 and February 2022. In search of case reports on HHM in breast cancer patients, five cases have been reported so far, which, in addition to the present three cases, are summarized in Table 1 [5–9]. In those eight cases, hypercalcemia rapidly developed, and it was severe (≥ 14 mg/dL) and symptomatic. Such clinical course of the HHM without bone metastasis in breast cancer patients was similar to that of HHM induced by other types of cancer [3]. As severe hypercalcemia may become fatal, physicians need to monitor serum calcium in breast cancer patients with visceral metastasis, and prompt treatment of hypercalcemia is of great importance. Extensive visceral metastases including the lung and the liver were present in seven of the eight cases, which suggests that a high tumor burden is necessary to develop HHM without bone metastasis.

**Table 1:** Summary of the HHM cases in breast cancer patients without bone metastasis

Case	Age at onset	Sex	Stage	Metastatic organ	DFI (m)	Subtype	From symptomatic onset to admission (m)	Maximum Ca (mg/dL)	Symptoms due to hypercalcemia	Treatment	Treatment duration (m)	Response to anticancer treatment	Outcome	References
1	53	F	Rec	Lung* Liver	120	ER + HER2 +	< 1	17.1	Yes	Hydration/calcitonin/bisphosphonates/furosemide	4	Poor	Dead	This report
2	46	F	Rec	Liver	32	ER + HER2–	< 1	15.8	Yes	Hydration/calcitonin/bisphosphonates/furosemide	< 1	Poor	Dead	This report
3	66	F	Rec	Lung* Liver	12	ER + HER2–	2	15.0	Yes	Hydration/calcitonin/bisphosphonates/furosemide	10	Good	Alive	This report
4	38	F	Rec	Lung	9	ER–	1	14.2	Yes	Hydration/calcitonin/glucocorticoids	1	Poor	Dead	[5]
5	49	F	Rec	Liver	33	ER + HER2–	1	15.8	Yes	Calcitonin/bisphosphonates/glucocorticoids	8	Good	Alive	[6]
6	46	F	IV	Liver LN	NA	ER– HER2 +	< 1	19.4	Yes	Hydration/calcitonin/bisphosphonates/glucocorticoids	14	Good	Alive	[7]
7	37	F	IV	Lung	NA	ER + HER2–	< 1	21.2	Yes	Hydration/calcitonin/bisphosphonates	13	Good	Alive	[8]
8	68	F	III B	LN	NA	ER + HER2–	NA	12.3	No	Hydration/calcitonin	6	Good (surgery)	Alive	[9]

*Rec* recurrence; *LN* lymph node; *DFI* disease-free interval; *ER* estrogen receptor; *HER2* human epidermal growth factor receptor-2; *NA* not applicable

*Complete remission at onset of hypercalcemia

HHM without bone metastasis in breast cancer patients is rare. The rare condition may be attributable to the property of PTHrP; PTHrP produced in breast cancer cells is associated with the development and progression of bone metastasis. The most important process in establishing bone metastasis is bone resorption induced by tumor cells. PTHrP secreted from breast cancer cells stimulates osteoblastic cells to upregulate receptor activator of NFκB ligand, which in turn promotes osteoclast formation, resulting in bone resorption [10]. The link between PTHrP and bone metastasis has been evidenced by experimental as well as clinical reports [11–14]. PTHrP is expressed in 60% of invasive breast tumors [15], suggesting that substantial proportion of hypercalcemia present in breast cancer patients with extensive bone metastasis, often recognized as LOH, is caused by multiple humoral factors including PTHrP, TNFα, and interleukins. Thus, it is plausible that HHM without bone metastasis is caused by the uncoupling of PTHrP-induced bone resorption and colonization of breast cancer cells in the bone. Elucidating the mechanism would help us develop a novel therapeutic approach preventing bone metastasis.

In accordance with HHM etiology, bone resorption inhibitors, such as calcitonin and bisphosphonates, are effective for HHM treatment. In addition, saline infusion for correcting dehydration and promoting calcium excretion is also important. Glucocorticoid helps reduce intestinal calcium absorption. These treatments are generally used in combination, which was the case in the eight cases of HHM without bone metastasis. The combination of those treatments was effective to rapidly decrease serum calcium level in patients with HHM without bone metastasis. For long-term control of serum calcium, treatment of breast cancer is essential. In the eight cases of HHM without bone metastasis, the favorable response to anticancer treatment led to the normalization of serum calcium for a long period. For example, in case 3, the treatment of HHM with bisphosphonate, hydration, and diuretics helped in the rapid decline of serum calcium, and the treatment of breast cancer with an anticancer drug, docetaxel, contributed to the sustained decrease in calcium concentration as shown in Fig. 1. To summarize, the multimodal therapy is of particular importance in the management of HHM without bone metastasis in breast cancer patients.

## Conclusions

Although HHM without bone metastasis in breast cancer patients is rare, its clinical features are similar to those in patients with other cancer types. Because HHM without bone metastasis develops rapidly, calcium monitoring and prompt treatment of hypercalcemia is vital. Also, successful treatment of metastatic breast cancer is a key to the long-term control of serum calcium.

## Data Availability

The dataset supporting the conclusion of this article is included within the article.

## Abbreviations

PTHrP: Parathyroid hormone-related protein
HHM: Humoral hypercalcemia of malignancy
LOH: Local osteolytic hypercalcemia
ER: Estrogen receptor
HER2: Human epidermal growth factor receptor-2
TNF: Tumor necrosis factor
CT: Computed tomography

## References

[1] Stewart AF (2005). Clinical practice. Hypercalcemia associated with cancer. N Engl J Med.

[2] Mirrakhimov AE (2015). Hypercalcemia of malignancy: an update on pathogenesis and management. N Am J Med Sci.

[3] Clines GA (2011). Mechanisms and treatment of hypercalcemia of malignancy. Curr Opin Endocrinol Diabetes Obes.

[4] Sternlicht H, Glezerman IG (2015). Hypercalcemia of malignancy and new treatment options. Ther Clin Risk Manag.

[5] Nagata N, Yasutomo Y, Kugai N, Matsuura Y, Akatsu T, Sugiyama K (1989). Parathyroid hormone-related protein and transforming growth factor activities in an extract from a breast cancer associated with humoral hypercalcemia of malignancy. Jpn J Clin Oncol.

[6] Bernard JD, Rimailho J, Pourrut JC, Hoff J, Becue J (1992). Hypercalcemia and breast cancer related to parathormone-like secretion by liver metastases. Gynecol Oncol.

[7] Tashiro H, Mashino K, Fujii K, Sakata H (2006). Report of a case of advanced breast cancer with no bone metastasis complicated by humoral hypercalcemia of malignancy. J Jpn Surg Assoc.

[8] Yamasaki K, Nakakuma T, Ueno S, Yuda T, Tsuru M, Tabei T (2020). A case of advanced breast cancer with hypercalcemia and no bone metastasis treated with multidisciplinary therapy. Gan To Kagaku Ryoho.

[9] Kuwahara K, Mokuno Y (2019). Synchronous bilateral breast cancer complicated by humoral hypercalcemia of malignancy—a case report. J Jpn Surg Assoc.

[10] Mundy GR (2002). Metastasis to bone: causes, consequences and therapeutic opportunities. Nat Rev Cancer.

[11] Thomas RJ, Guise TA, Yin JJ, Elliott J, Horwood NJ, Martin TJ (1999). Breast cancer cells interact with osteoblasts to support osteoclast formation. Endocrinology.

[12] Guise TA, Yin JJ, Taylor SD, Kumagai Y, Dallas M, Boyce BF (1996). Evidence for a causal role of parathyroid hormone-related protein in the pathogenesis of human breast cancer-mediated osteolysis. J Clin Investig.

[13] Mundy GR, Martin TJ (1982). The hypercalcemia of malignancy: pathogenesis and management. Metabolism.

[14] Clohisy DR, Ramnaraine ML (1998). Osteoclasts are required for bone tumors to grow and destroy bone. J Orthop Res.

[15] Southby J, Kissin MW, Danks JA, Hayman JA, Moseley JM, Henderson MA (1990). Immunohistochemical localization of parathyroid hormone-related protein in human breast cancer. Cancer Res.

